# Enhanced hypoglycemic effect of biotin-modified liposomes loading insulin: effect of formulation variables, intracellular trafficking, and cytotoxicity

**DOI:** 10.1186/1556-276X-9-185

**Published:** 2014-04-16

**Authors:** Xingwang Zhang, Jianping Qi, Yi Lu, Xiongwei Hu, Wei He, Wei Wu

**Affiliations:** 1 School of Pharmacy, Fudan University, Key Laboratory of Smart Drug Delivery, Ministry of Education and PLA, Shanghai 201203, People's Republic of China; 2 Division of Pharmaceutics, College of Pharmacy, Jinan University, Guangzhou 510632, People's Republic of China

**Keywords:** Insulin, Biotin, Liposomes, Hypoglycemic effect, Oral, Ligand-mediated, Endocytosis, Cytotoxicity

## Abstract

Peroral protein/peptide delivery has been one of the most challenging, but encouraging topics in pharmaceutics. This article was intended to explore the potential of biotin-modified liposomes (BLPs) as oral insulin delivery carriers. By incorporating biotin-DSPE into the lipid bilayer, we prepared BLPs using reverse evaporation/sonication method. We investigated hypoglycemic effects in normal rats after oral administration of BLPs, and the possible absorption mechanism by a series of *in vitro* tests. The relative pharmacological bioavailability of BLPs was up to 11.04% that was as much as 5.28 folds of conventional liposomes (CLPs). The results showed that the enhanced oral absorption of insulin mainly attributed to biotin ligand-mediated endocytosis. The results provided proof of BLPs as effective carriers for oral insulin delivery.

## Background

With the advent of biotech epoch, more and more proteins and peptides become available for clinical treatment, such as growth hormone [1], calcitonin [2], and octreotide [3]. Nevertheless, due to short half-life in the blood circulation, it is inevitable to take the medications subjected to multi-dosage over a long time for chronic diseases. Insulin, a protein secreted by the β cells of the pancreas, is one of the most important therapeutic agents for insulin-dependent (type I) and deteriorative insulin-independent (type II) diabetes mellitus [4], and commonly administered subcutaneously; however, besides pain, which may bring about unwanted complications, e.g. allergic reactions, hyperinsulinemia, insulin lipodystrophy around the injection site [5]. Problems encountered with insulin injection vitalize the demands to develop alternative delivery systems.

However, to achieve effective oral delivery of insulin, several barriers like instability, gastrointestinal enzymatic degradation, and poor membrane permeability, etc., should be overcome beforehand [6]. Various delivery strategies, especially those based on nanoscaled delivery systems, have been explored to enhance the oral delivery of insulin, including microemulsions [7], nanospheres [8], polymeric nanoparticles [9,10], niosomes [11], and liposomes [12-14]. However, the state of the art indicates that there seems to have reached a bottleneck in terms of oral bioavailability enhancement of insulin. It is highly recommended to explore novel strategies to ameliorate the performance of nanoscaled drug delivery systems. As known, receptor-mediated endocytosis, a process of internalization of extracellular molecules during which a binding occurs between the molecules and the receptors, is an important absorption mechanism for substances like proteins, hormones, growth factors, and fatty nutrients [15]. In the intestinal cavity, there are a variety of receptors expressing on the membrane of enterocytes such as vitamins, transferrin, amino acids, sugars, and bile salt receptors [16]. Targeted drug delivery to absorptive epithelia by receptor-mediated endocytosis has emerged as a prominent means to improve oral delivery of drugs [17]. Vitamins as ligands, which can specifically bind to enterocytic receptors, have been extensively studied for the oral delivery of poorly permeable molecules [18-22].

Biotin receptors that distribute in the small intestine and partially in the colon are responsible for the essential absorption of biotin by nonspecific receptor-mediated endocytosis [23]. Additionally, biotin plays an important role in maintaining the homeostasis of blood glucose [24]. Improved cellular permeability and higher hypoglycemic effect after oral administration of biotin-conjugated glucagon-like peptide-1 has been observed [25]. Biotin-modified vehicles have been investigated for nonparenteral delivery of active ingredients [26-29]. Our previous report has also proved that biotin-modified liposomes (BLPs) have ability to improve the oral delivery of insulin, and studied the uptake and transport mechanisms in the gastrointestinal tract [30]. However, particular enhanced absorption mechanisms and cytotoxicity of BLPs are not clear enough.

Herein, we performed several experiments to further probe the oral absorption mechanism of BLPs based on previous studies [30] as well as the cytotoxicity thereof. We evaluated hypoglycemic effects of BLPs of various particles, or with different amounts of biotin-DSPE using normal rats. Meanwhile, the influence of BLPs on tight junctions and internalization process was further investigated by Caco-2 cells.

## Methods

### Materials

Porcine insulin (29 IU/mg) was provided by Jiangsu Wanbang Biochemical Pharmaceutical Co, Ltd (Xuzhou, China). Soybean phosphatidylcholine (SPC, Lipoid S75), cholesterol (CH), and 1, 2-distearoyl-sn-glycero-3-phosphatidyl ethanolamine (DSPE) were supplied by Lipoid (Ludwigshafen, Germany). Fluorescein isothiocyanate (FITC) and biotin were purchased from Sigma (Shanghai, China). Sephadex G-50 was obtained from Pharmacia (Shanghai, China). Deionized water was prepared by a Milli-Q purification system (Molsheim, France). HPLC-grade acetonitrile was provided by Merck (Darmstadt, Germany). All other chemicals were of analytical grade and used as received.

### Preparation of BLPs

SPC, biotin-DSPE (synthesized according to previous report [30]), and cholesterol were dissolved in absolute ether to prepare the organic phase, into which the aqueous phase, insulin citric acid-Na_2_HPO_4_ buffer solution (pH 4.0, if not specified otherwise), was added dropwise following ultra-sonication to prepare the W/O emulsion. The organic solvent in the emulsion was then evaporated toward a rota-evaporator under 0.05- to 0.06-MPa pressure at a rotating speed of 50 rpm at 30°C until glutinous gel formed. Afterwards, citric acid-Na_2_HPO_4_ buffer with pH 3.8 was instilled to hydrate the lipidic gel until a homogeneous dispersion was formed. Finally, the dispersion was homogenized through an ultrasound cell cracker (SCIENTZ IID, Scientz Biotechnology, Ningbo, China) to obtain insulin-loaded BLPs. To prepare insulin-loaded conventional liposomes (CLPs) and blank liposomes, same procedures were followed as described above.

The particle size of liposomes was measured by dynamic light scattering using Zetasizer Nano ZS (Malvern, Worcestershire, UK) at 25°C. The morphology of the liposomes was characterized by transmission electron microscopy (TEM). Briefly, liposomes were dripped onto a piece of copper grid and negatively stained with 1% (*w*/*v*) phosphotungstic acid for 1 min at room temperature. The stained nanoparticles allowed to dry at ambient condition and then were observed with TEM (JEM-1230, Tokyo, Japan) at an acceleration voltage of 120 kV.

### Entrapment efficiency

Entrapment efficiency of insulin-loaded liposomes was determined by molecular exclusion chromatography using Sephadex G50 column to separate the free insulin from liposomes [31]. Briefly, liposome samples were added into the top of column and eluted with the same buffer to liposomes hydration. The eluted fraction of insulin-enveloped liposomes was analyzed by HPLC according to the reported procedure [32]. The entrapment efficiency (EE) was defined as the ratio of liposome-enveloped insulin (insulin_env_) to total insulin (insulin_tot_), namely EE (%) = Insulin_env_/Insulin_tot_ × 100%.

### Hypoglycemic effect in normal rats

Normal rats (SD, 220 ± 20 g), supplied by Shanghai Laboratory Animal Resource Center, were applied to the evaluation of the hypoglycemic effect. The rats were fasted for 12 h ahead of administration, but allowed free access to water during the sampling. All animal experiments were conducted in accordance with the approval of Experimental Animal Ethical Committee of Fudan University. The intragastric (*i.g.*) dose of liposomes was equivalent to 20 IU/kg of insulin, while the subcutaneous (*s.c.*) dose of insulin solution as reference was set to 1 IU/kg. Blood samples (150 μL) were collected from the tail vein at specific intervals into heparinized tubes, and immediately centrifuged at 5,000 g for 5 min to gather the plasma. Blood glucose was determined in triplicate by the glucose oxidase method using Glucose GOD-PAD kit (Rongsheng Biotech, Shanghai, China). Besides, we investigated the influence of particle size, biotin-DSPE proportion and dose of liposomes after oral administration on the hypoglycemic effect in rats. The relative bioavailability was calculated based on the pharmacological activity following the equation below:

$$\mathrm{PA}\left(\%\right)=\frac{{{\mathrm{AAC}}_{0\;\mathrm{to}\;12h}}_{\left(i.g.\right)}\times {\mathrm{Dose}}_{\left(s.c.\right)}}{{\mathrm{AAC}}_{0\;\mathrm{to}\;12h\left(s.c.\right)}\times {\mathrm{Dose}}_{\left(i.g.\right)}}\times 100\%$$

where AAC was the overall area above the plasma glucose levels *vs.* time curve calculated by the trapezoidal method using a reference line obtained from the base control.

### Absorption mechanism studies

In order to continue to investigate the absorption mechanism, the changes of the transepithelial electrical resistance (TEER) of Caco-2 cell monolayers after incubation with insulin-loaded liposomes and the intracellular trafficking of BLPs were determined. The methods of cell culture, Caco-2 cell monolayer construction and the synthesis of fluorescent probes were the same as the previous report [30].

To determine the TEER, the well-cultured Caco-2 cell monolayers were incubated with insulin preparations, and the TEERs of Caco-2 cell monolayers were determined at different times by a Millicell Electrical Resistance System equipped with STX-2 electrodes (Millipore, Bedford, MA, USA).

To study the intracellular trafficking of BLPs, cells were cultured on coverslips for 5 days prior to testing. For endosome investigation, the cells were treated with rhodamine-labeled BLPs for 2 h. Then, the cells were continued to be incubated with Rabbit polyclonal antibody Rab5 (ab18211, Abcam, UK) and Mouse monoclonal Rab7 (ab50533, Abcam, UK) overnight at 37°C followed by the addition of a secondary antibody FITC-goat anti-rabbit IgG to identify the early and later endosomes. For lysosome investigation, the medium containing LysoTracker® Red DND-99 g was added into the cells beforehand to label the lysosomes for 2 h. Subsequently, the cells washed with PBS were incubated with FITC-labeled insulin (FITC-ins) loaded BLPs for another 2 h. Finally, the media were removed from the cells and the co-localizations of BLPs with cytoplasmic vesicles were observed by confocal laser scanning microscopy (CLSM).

### *In vitro* cytotoxicity evaluation of liposomes

The cytotoxicity of the liposomes was examined by assessing the viability and apoptosis of Caco-2 cells in the presence of different concentrations of liposomes. The viability of the cells was measured using the MTT assay. Caco-2 cells were cultured for 48 h and rinsed with PBS three times, into which liposomes with various lipid levels were introduced. After incubation for 5 h at 37°C, the MTT solution (20 μL, 5 mg/mL) was added to each well holding cells and continued to incubate for 4 h. DMSO (200 μL) was added to each well to dissolve completely the internalized purple formazan crystals when the medium and excess MTT were removed. UV absorbance of each well was tested at a wavelength of 490 nm. Cell viability was calculated from the ratio between the number of cells treated with the liposomes and that of the control (blank) following the equation: Cell viability (%) = (*A*_tri_/*A*_con_) × 100%, where *A*_tri_ was the absorbance intensity of the cells treated with liposomes, and *A*_con_ was the value treated with PBS. The cells treated with culture medium served as 100% cell viability.

To assess the effect of liposomes on cell apoptosis, liposomes with different lipid concentrations were added into the cells and incubated for 4 h. The state of apoptosis were analyzed by detecting the phosphatidylserine (PS) translocation of cell membranes using annexin V-FITC and PI double staining in order to differentiate apoptotic cells from necrotic cells. The analysis was conducted by flow cytometry with an annexin V-FITC/PI apoptosis detection kit (Keygen Biotech, Nanjing, China). Each sample was repeated three times using 10^5^ cells per test. The cells treated with PBS were set as the control.

## Results and discussion

### Preparation and characterization of BLPs

Formulation variables influence the physicochemical properties of insulin-loaded liposomes such as entrapment efficiency and particle size [32,33]. It is of high importance to effectively entrap insulin into liposomes so as to reduce the bulk dosage and avoid waste of drug. The main variables including lipid/cholesterol ratio, drug/lipid ratio, the buffer pH upon hydration, and phase ratio in preparing W/O emulsion were optimized to obtain liposomes with high insulin entrapment efficiency and suitable particle size.

**Figure 1:**
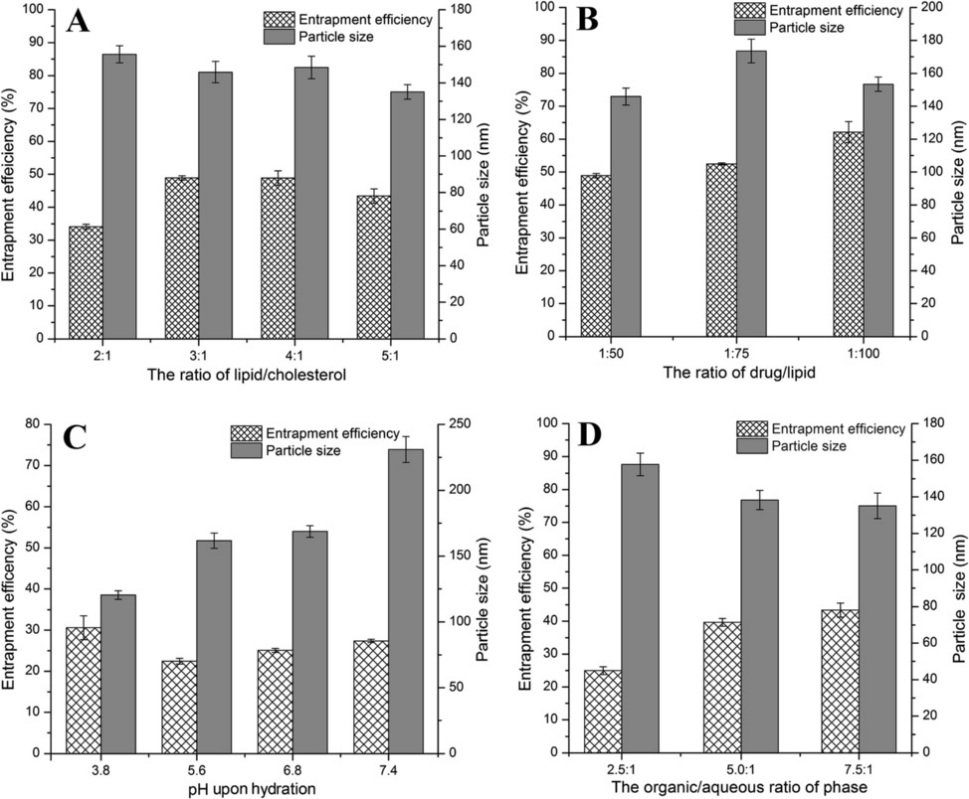
**The effects of formulation variables on entrapment efficiency and particle size. (A)** Ratios of lipid/cholesterol and **(B)** drug/lipid, **(C)** pH upon hydration, and **(D)** organic/aqueous ratio of phase.

Figure 1 shows the effects of preparative variables on the entrapment efficiency and particle size. The presence of cholesterol exerts significant influence on the properties of the lipid bilayers of the liposomes. It is known that the addition of cholesterol to lipid bilayers decreases its permeability to water [34]. Suitable lipid/cholesterol ratio will accommodate more insulin molecules and generate liposomes with desirable membrane fluidity, which are helpful to prevent the leakage of insulin from the internal aqueous compartments. The liposomes with a lipid/cholesterol ratio of 3/1 or 4/1 produced higher insulin entrapment (Figure 1A). Considering the factors that influenced drug entrapment and resistant permeability to water, a lipid/cholesterol ratio of 3/1 seemed to be more promising. The effect of drug/lipid ratio on the entrapment efficiency is shown in Figure 1B, from which we could see that the entrapment efficiency increased as the lipid content increased. Generally, high proportion of lipid in liposomes can generate more space to host more insulin molecules. Figure 1C showed that the buffer solution of pH 3.8 used for hydration was most suitable to prepare liposomes. Lower entrapment efficiencies were obtained around the isoelectric point of insulin (pH 5.3 to 5.4), which may be attributed to the loss of insulin because of reduced solubility. The particle size of liposomes increased as the pH increased owing to the change of surface charge. In general, natural phospholipids such as soybean or egg lecithins are negatively charged. When pH goes up, the charge density of phospholipids will raise correspondingly, which results in more electrostatic repulsion that is unfavorable to form small liposomes. Higher organic-aqueous phase ratio resulted in higher entrapment efficiency as observed from Figure 1D because increasing the organic phase was beneficial to the formation of fine emulsions, which would lead to fine insulin dispersion in the mixed lipids.

**Figure 2:**
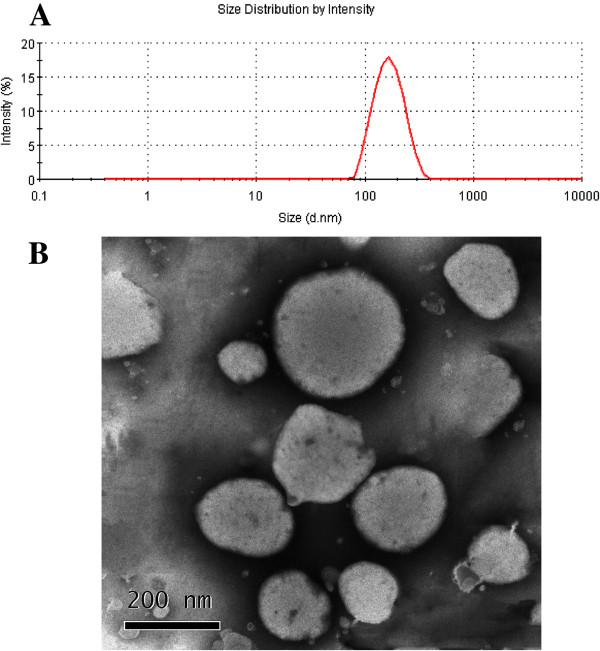
Particle size (A) and TEM micrograph of BLPs (B).

The mean particle size of BLPs prepared under optimal conditions were about 160 nm and presented a unimodal distribution (Figure 2A). The morphology of BLPs was near-spherical as observed by TEM (Figure 2B), though the liposomes looked somewhat irregular in the TEM as a consequence of membranous deformation and dehydration in sample preparation.

### Factors influencing hypoglycemic effect of BLPs

**Figure 3:**
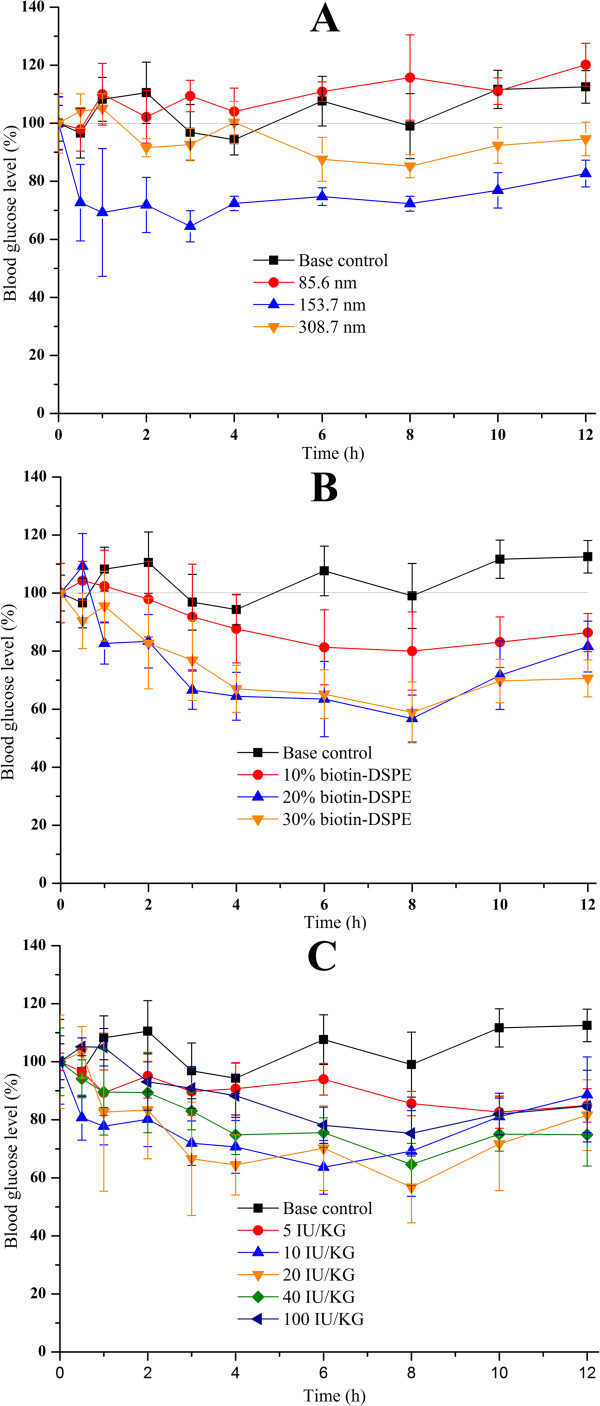
**Profiles of blood glucose in rats after oral administration of insulin-loaded BLPs.** Different particle sizes (**A**, 20 IU/kg), biotin-DSPE proportions (**B**, 20 IU/kg), and doses orally given **(C)**.

The performance of BLPs in decreasing the blood glucose of rats was affected by a variety of factors. The influences of particle size of liposomes, biotin-DSPE proportion in liposomes, and doses orally given on hypoglycemic effect are shown in Figure 3. As shown in Figure 3A, liposomes with a diameter about 80 nm almost did not pose a declined blood glucose. The negative result may be the cause of fragile structure that easily destroyed by harsh GI environment featured by digestive enzymes and low pH. However, a significant hypoglycemic effect was observed when the rats were orally administrated of liposomes of 153.7 nm, the maximal decline of blood glucose level was up to 38.4%. This enhanced pharmacological action by BLPs at 150 nm around may be attributed two facets: (i) improved stability relative to liposomes with a smaller diameter and (ii) facilitated uptake through intestinal epithelia, especially by receptor-mediated endocytosis. With the increase of diameter of liposomes, although the physical stability was further strengthened, the hypoglycemic effect of BLPs not only fail to raise but decrease, which may be caused by larger particle size that was unfavorably absorbed by epithelia, particularly through vesicle-mediated transport such as by clathrin-coated pits.

The blood glucose profiles of rats after oral administration of liposomes with different biotin-DSPE levels are shown in Figure 3B. Liposomes with 10% biotin-DSPE, to some extent, produced the decline of blood glucose of rats after dosing, whereas more obvious downtrends occurred in the rats those were given of liposomes with biotin-DSPE above 20%. However, there was no significant difference between the liposomes composed of 20% and 30% biotin-DSPE in terms of hypoglycemic effect. The hypoglycemic effect produced by liposomes with 10% biotin-DSPE weaker than that of liposomes with more biotin-DSPE may be as a consequence of relatively weak stability rather than the insufficiency of ligand amount, because DSPE that possesses a high phase transition temperature could reinforce the rigidity of liposomes if more biotin-DSPE was incorporated into lipid bilayer. However, since there was not much difference in the stability of liposomes, increasing the amount of biotin-DSPE is not helpful to the absorption of liposomes, which could also be deduced from the results of hypoglycemic effect using liposomes containing 20% and 30% biotin-DSPE that presented similar profiles of blood glucose decline.

The changes in the blood glucose level of rats after oral administration of different doses of BLPs are displayed in Figure 3C. Below the dose of 20 IU/kg, the hypoglycemic effect of BLPs increased with the increase of oral dose, presenting a dose dependency. At high doses above 20 IU/kg, however, the *in vivo* hypoglycemic effects of BLPs were maintained in the analogous level and seemingly arrived to a plateau. The phenomenon that the hypoglycemic effect of BLPs linearly correlated with the dose given at low doses and expressed nonlinearity at high doses may be ascribed to the saturability of biotin receptors on enterocytes.

### Enhanced hypoglycemic effect of insulin *via* BLPs

**Figure 4:**
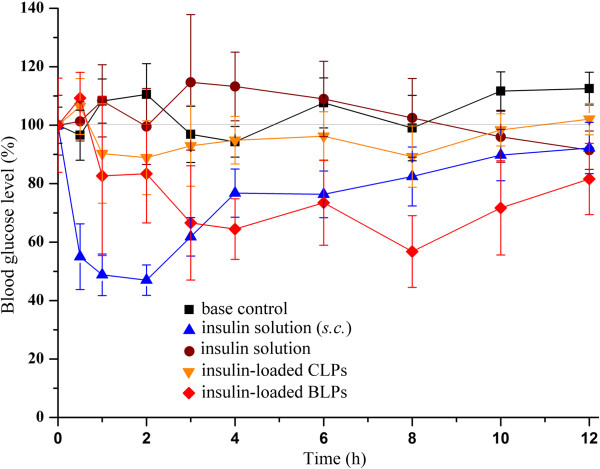
**Blood glucose levels in rats after administration of insulin solution and insulin liposomes (the mean ± SD, *n =6*).**

The hypoglycemic effects in normal rats are shown in Figure 4. Subcutaneous (*s.c.*) injection of insulin solution produced rapid blood glucose decrease to about 50% of normal level in the first 2 to 3 h, and then quickly rebounded to normal level. Due to significant GI digestion, oral administration of free insulin showed little hypoglycemic effect. The blood glucose fluctuated, possibly posed by force-feeding stress, within the initial 3 h but maintained at the normal level thereafter. Oral CLPs just resulted in a slight drop in blood glucose level, though oral administration of BLPs produced gradual glucose decrease to about 60% of the normal level at 8 h. However, the blood glucose of rats discontinued to decrease owing to the compensatory mechanism that could actuate the decomposition of glycogen to compensate for the loss of blood glucose. The relative pharmacological bioavailability of BLPs, calculated by the trapezoidal method, was 11.04% with *s.c.* insulin as the reference, for CLPs just 2.09%. This result highlighted the effectiveness of biotin modification on the absorption of insulin-loaded liposomes.

### Potential absorption mechanism

In previous studies, enhanced cellular uptake and internalization by specific clathrin-mediated endocytosis was found in terms of BLPs, and the enhanced performance had nothing to do with the opening of intercellular tight junctions [30]. To further interpret the absorption mechanism of BLPs, we executed another several cell experiments to deepen the prior results.

**Figure 5:**
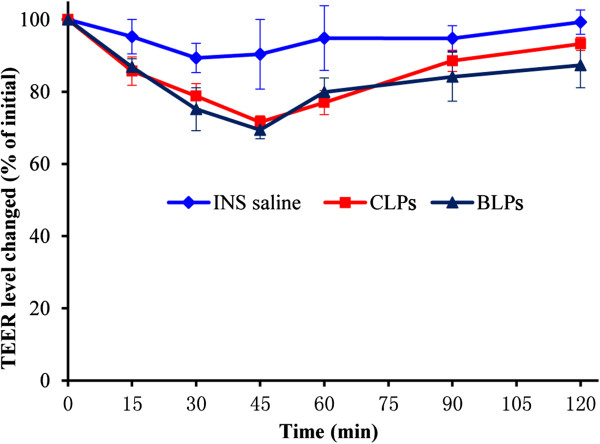
**Effects of insulin saline and insulin-loaded liposomes on TEER of Caco-2 cell monolayers.** Group treated with DMEM as reference.

In order to clarify whether the paracellular pathway responsible for the enhanced oral delivery of BLPs, we investigated the influence of BLPs on tight junctions by determining the TEER of Caco-2 cell monolayers. Figure 5 shows the TEER changes of Caco-2 cell monolayers after incubation with insulin saline and insulin-loaded liposomes. In comparison with insulin saline, two kinds of liposomes merely caused a transient decline of TEER of Caco-2 cell monolayers upon incubation, whereas the extents in decline were relatively low, which may result from the alternation of media that motivated the increased cell activity. After 45 min, the TEER of Caco-2 cell monolayers was restored to the initial level, while a similar process happened in the group treated with insulin saline. However, there was no significant difference between BLPs and CLPs in the alteration of TEER, indicating that the enhanced oral absorption of BLPs was not caused by the opening of tight junctions.

**Figure 6:**
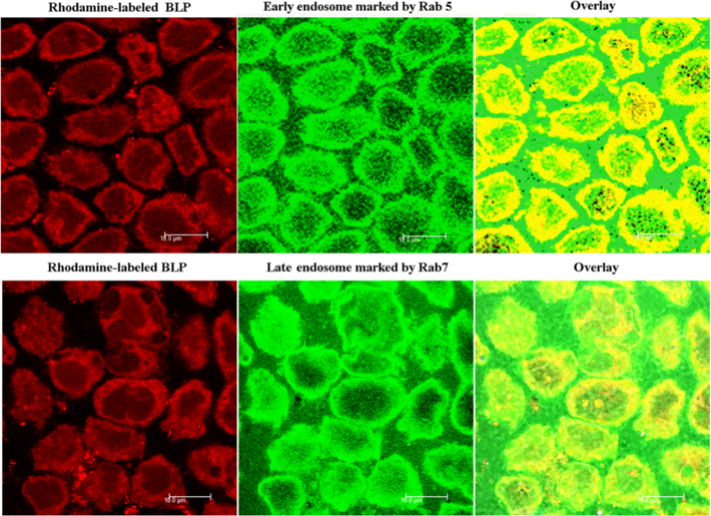
**CLSM observation of the co-localization of Rhodamine-labeled BLPs into endosomes.** The co-localizations of BLPs with Rab5/Rab7 are presented in yellow fluorescence.

**Figure 7:**
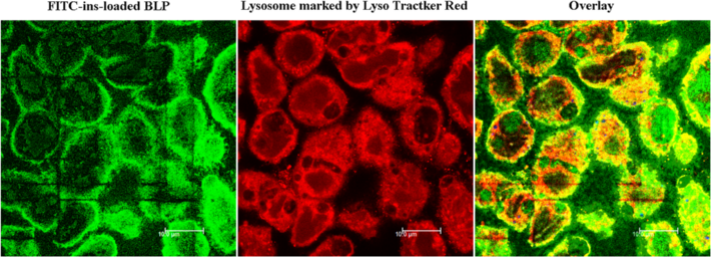
**CLSM observation of the co-localization of FITC-ins-loaded BLPs into lysosomes.** The yellow color in the overlay denotes the co-localization of lysosomes with BLPs.

As the best knowledge known, receptor-mediated endocytosis is a process of internalization of extracellular molecules during which vesicles, for example endosomes and lysosomes, are formed, which is highly characteristic for receptor-mediated endocytosis [35]. The co-localizations of BLPs with endosomes by CLSM observation are shown in Figure 6. The yellow areas, typifying the co-localization, were found to locate either in early endosomes or in late endosomes after incubation with BLPs, clearly stating that BLPs after being internalized into cells is experiencing membrane-associated protein-coated pit invagination to form endosomes. Furthermore, the co-localization of BLPs was mainly distributed over the boundary area in the early endosomes; however, in the late endosomes, the co-localization had a tendency of transferring the cytoplasm inward, indicating the disassociation of coating proteins from the invaginated vesicles. Following the confirmation of endosome transport, we further investigated the intracellular trafficking of BLPs using Lyso Tracter® Red, a tracing marker of acidic organelles. The co-localization of BLPs with lysosomes is presented in Figure 7. It could be seen that FITC-ins-loaded BLPs entered into the liposomes and the lysosomes were explicitly labelled into red. An overlay (yellow) of green representing FITC-ins-loaded BLPs and red representing lysosomes was observed, which indicated that the intracellular trafficking of BLPs after the formation of late endosomes experienced the transport from late endosomes to lysosomes. The abovementioned facts provided solid proof that the oral absorption of BLPs was facilitated by biotin receptor-mediated endocytosis.

### Cytotoxicity of BLPs

**Figure 8:**
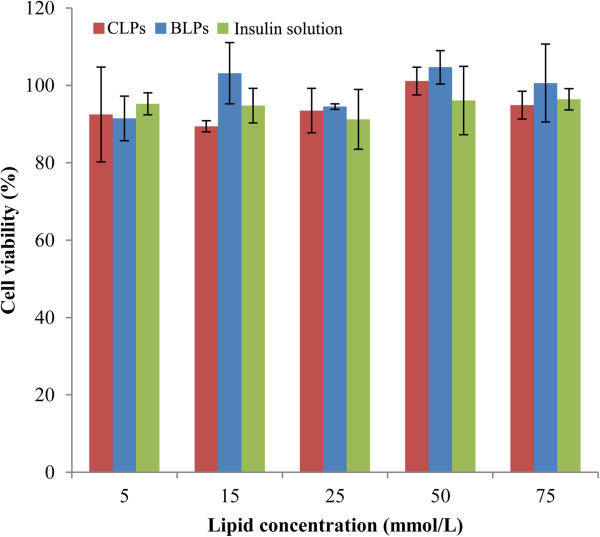
**Cell viability of Caco-2.** Incubated with BLPs or CLPs at different lipid concentrations as well as insulin saline for 4 h. (*n* = 3).

**Figure 9:**
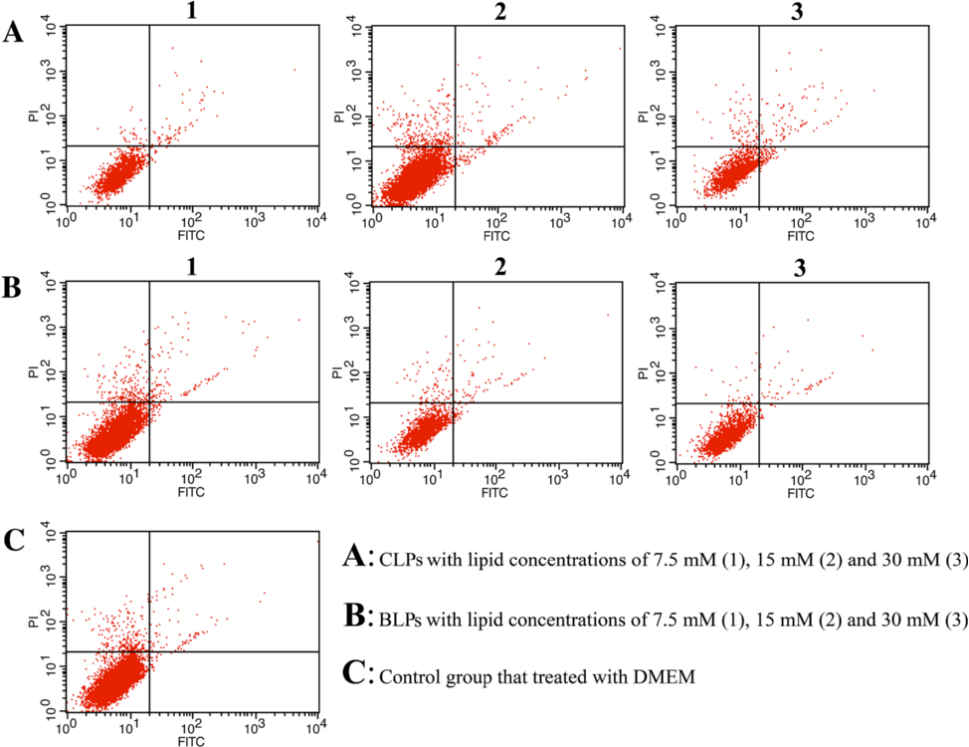
**Distribution of cells in different apoptotic stages treated with liposomes at different lipid concentrations for 4 h.** Collection of annexin V signals as FL1 and propidium iodide (PI) signals as FL2.

Regarding the biomaterial of biotin-DSPE, we have not much information about its toxicity; hence, it is necessary to evaluate the cytotoxicity for the sake of oral safety. Figure 8 shows the cell viability of Caco-2 cells after incubation with insulin preparations. No obvious cytotoxicity was observed for insulin solution or insulin-loaded liposomes after a 4-h incubation when compared to the negative control. The cell viability of the insulin solution group still remained above 95%, and two liposomes had negligible difference in cell viability relative to insulin solution under various lipid concentrations. Besides, the cytotoxicity of BLPs was on close level in comparison with CLPs, indicating that the biotinylation of liposomes did not bring extra toxicity. Furthermore, the desirable biocompatibility could also be judged from the result of apoptosis of Caco-2 cells (Figure 9). The effects of BLPs and CLPs at three lipid concentrations on the apoptosis were relatively insignificant relative to the negative control. In quadrant 4 (Q4) betokening the early apoptotic cells, there were no positive signals detected either for BLPs or CLPs, declaring that liposomes, whether being biotinylated or not, did not significantly cause the apoptosis of cells. Although some late apoptotic cells were observed in Q2, they may come from the necrotic cells as a result of natural mortality of cells rather than the apoptosis induced by liposomes. The results indicated that biotin-modified liposomes had a good oral safety for insulin delivery.

## Conclusion

This research provided insight into the potential of biotinylated liposomes as novel nanocarriers for oral insulin delivery. Liposomes prepared under optimal conditions can effectively entrap insulin into the inner aqueous cavity and improve the stability of transportation through the GI tract. By biotinylation, the GI absorptive feature of liposomes was notably enhanced. Significant hypoglycemic effect was observed in rats in comparison with CLPs after oral administration of BLPs, especially using liposomes with a particle size about 150 nm. The enhanced oral delivery of insulin was mainly ascribed to ligand-mediated endocytosis by targeting to biotin receptor on enterocytes.

## Competing interests

The authors declare that they have no competing interests.

## Authors’ contributions

WW and JQ had conceived and designed experiments. XZ and YL carried out synthesis and characterization of biotin-DSPE. XZ, XH, and WH performed animal experiments. XZ and JQ performed cell experiments. XZ, WW, and JQ wrote the manuscript. All authors read and approved final manuscript.

## Authors’ information

XZ, XH, and WH are Ph.D students at Fudan University. JQ holds a lecturer position at Fudan University. YL and WW hold associate professor and professor position at Fudan University, respectively.
